# Finite Element Simulation of the Laser Shock Peening Process on 304L Stainless Steel

**DOI:** 10.3390/ma18132958

**Published:** 2025-06-23

**Authors:** Mayur B. Wakchaure, Manoranjan Misra, Pradeep L. Menezes

**Affiliations:** 1 Department of Mechanical Engineering, University of Nevada, Reno, NV 89557, USA; 2 Department of Chemical and Materials Engineering, University of Nevada, Reno, NV 89557, USA

**Keywords:** laser shock peening, finite element analysis, residual stresses

## Abstract

This study investigates the effects of Laser Shock Peening (LSP) on residual stress distribution and surface deformation using a Finite Element Method (FEM) model. LSP is a surface treatment process that generates compressive residual stress by applying high-energy laser pulses over nanosecond timescales. The study aims to analyze the impact of key parameters, specifically laser spot overlap rate and power density, on the induced residual stress and surface deformation. A Design of Experiment (DOE) approach was used to systematically vary these parameters. These simulations were performed using the ANSYS Explicit Dynamics FEM with a Johnson–Cook material model to capture the nonlinear constitutive behavior. The research analyzes the distribution of residual stress and surface deformation caused by LSP. Increasing laser spot overlap and power density leads to higher compressive residual stress and surface deformation, revealing two distinct behavioral outcomes: either deep compressive stress with minimal deformation or a transition from compressive to tensile stress followed by significant surface deformation and a subsequent return to compressive stress. The results demonstrate strong agreement with existing experimental data presented in the literature. This study contributes novel insights into the interaction between LSP parameters and their effects on material properties, with implications for understanding LSP techniques in practical applications. The triangular pulse model and dual-overlap analysis offer a novel simulation strategy for optimizing LSP parameters in stainless steel.

## 1. Introduction

**Figure 1:**
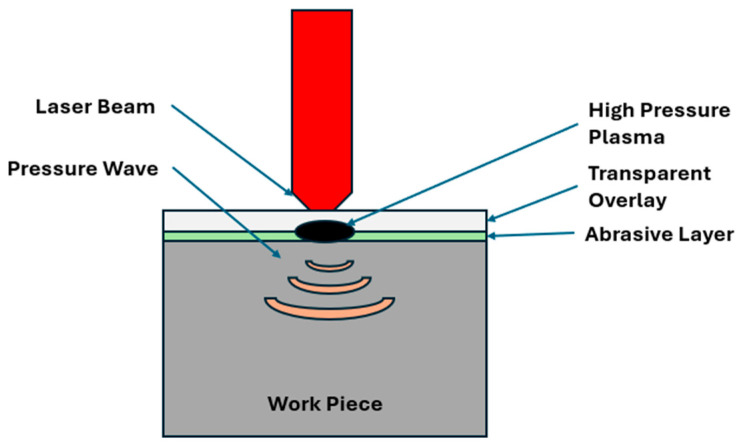
Schematic of the laser shock peening process.

Surface treatment of metallic materials can significantly extend their service life at a minimal cost. In 1962, research conducted by Askar’yan and Moroz provided significant insights into the effect of high-intensity lasers on materials, particularly on a microscopic scale [1]. These experiments led to the development of stress fields in surface layers, thereby enhancing the mechanical properties of the materials. Over the following decade, this process was refined and became known as Laser Shock Peening (LSP) [2]. The physics behind LSP can be understood from Figure 1.

The main principle of mechanically based surface treatment processes is to create a plastically deformed layer on the metallic material’s surface [2,3]. This plastic deformation introduces compressive residual stress in the surface and subsurface layers, which create resistance for fatigue crack propagation [3], tribo-corrosion [4], wear [5], corrosion [6,7,8], fretting-corrosion [9], and stress corrosion cracking [10]. The level of plastic deformation, as well as the magnitude and depth of the compressive residual stress, varies depending on the surface treatment method. This deformation often comes with parallel microstructural changes, which improve the material’s mechanical properties [8]. Current research is focused on surface treatments that produce optimal compressive residual stress, grain refinement, and minimal deformation, particularly in 304L stainless steel. LSP is one such surface modification technique that offers extensive control over parameters to induce deep, plastically deformed surfaces with compressive stress [2].

The LSP process can be carried out using a variety of laser sources with different wavelengths to deliver the required energy to the material’s surface. Specifically, the Nd-pulsed laser is one of reliable lasers for delivering energy pulses in the range of GW/cm^2^ [10]. The LSP process is further refined by incorporating a confinement medium and overlay coatings on the material. While black tape or black paint are commonly used as ablative layers in LSP, other materials such as aluminum foil, graphite coatings, or plastic films can also be employed, depending on the material system and desired ablation characteristics [8]. This high pressure creates a shockwave and creates an impact on the material’s surface. Usually, water is used as confinement medium to restrict the pressure from the plasma, directing the shockwave into the material to induce plastic deformation and grain refinement. When the shockwave exceeds the Hugoniot Elastic Limit (HEL) of the material, plastic deformation occurs at the surface with a high strain rate for a short duration [2]. During LSP, the material experiences strain rates on the order of 10^6^ per second. Given the complexity of the LSP process and the mechanical and metallurgical changes that occur post-process, understanding the deformation mechanisms are challenging. Complex-shaped components can also be treated using laser oblique shock peening [11]. Higher laser intensity creates stronger shock waves, which induce deeper compressive residual stresses and enhance fatigue resistance. The increased energy density promotes greater plastic deformation of the material, leading to more significant microstructural changes. However, if the intensity is too high, it can cause surface damage or melting, compromising the material’s integrity and potentially causing issues like micro-cracking or surface roughness. On the other hand, lower laser intensity generates milder shock waves that may not penetrate as deeply, resulting in more modest improvements in material properties. This approach can be advantageous for treating delicate or thin materials where excessive energy could cause damage, but it may not achieve the same level of fatigue resistance or hardness as higher intensities [12,13]. A recent study on optimizing laser overlap ratios has revealed important insights into stress dynamics, showing that increased overlap between laser beams correlates with higher stress levels, including heightened tensile stresses on the surface. This correlation underscores the significance of the overlap ratio in determining the magnitude of stress experienced by the material, which is vital for optimizing laser-based processes and addressing potential structural concerns. The overlapping rate is particularly influential in residual compressive stress fields, affecting the consistency and thickness of the stress layer. Higher overlap typically results in a more uniform and thicker residual compressive stress field, enhancing material properties like fatigue and wear resistance. Understanding this relationship is essential for engineering practices involving material strengthening or surface modification [2,14,15,16].

Finite Element Analysis (FEA) of LSP has shown that the depth of compressive stress and plastic strain induced by laser pulses can be controlled by adjusting the laser spot size and the percentage of overlap between laser spots [15]. Multiple laser parameters and their combinations can be used to determine the number of experiments needed to assess the impact of different parameter combinations. FEA simulations of parallel LSP processes with varying laser spot sizes, intensities, and spacing based on design of experiment (DOE) reveal that increasing laser spot size reduces energy attenuation, while overlapping laser spots promotes more uniform stress distribution compared to increasing spot spacing [15,17,18,19]. The optimal combination of parameters is necessary for achieving the required residual stress with minimal surface distortion. A single set of laser parameters cannot produce the same effect across all materials, necessitating proper investigation for each material.

The effectiveness of LSP is influenced by various laser parameters, and variations in these parameters can significantly affect different materials based on their properties. To measure effectiveness in physical components requires advanced characterization techniques such as XRD and real-time monitoring systems, which tend to be costly [20]. In this context, optimizing process parameters and performing simulations are essential to analyze outcomes for specific parameter sets on the material. Researchers have conducted LSP simulations using precise material models that closely resemble experimental conditions, allowing for a deeper understanding of how laser parameters impact residual stress profiles [21,22,23]. FEA of LSP has been conducted by various researchers to explore its effects on different materials [2]. However, one study [7] reveals that there are relatively few comprehensive FEA studies specifically focused on stainless steel materials like SS 304L. This gap in the literature makes SS 304L an ideal material for further simulation. This simulation is compared with the actual results of experimentation conducted by our group [8], incorporating a new approach for laser energy input in the form of a triangular wave to enhance the simulation process. The results were observed along the depth to analyze the residual stresses, allowing for a detailed comparison between the simulation and experimental values.

This research aims to propose an optimal set of laser parameters for Stainless Steel 304L (SS 304L) to achieve maximum residual stress with minimal surface distortion using an Ansys Explicit Dynamics FEA simulation. The combined effects of different overlap rates and power densities were analyzed, while keeping the spot size, number of shots, and shot sequence fixed, to evaluate their influence on residual stress distribution and surface distortion in SS 304L. In the simulation work, two laser spots were overlapped at 30% and 50%, combined with four laser intensities: 3, 5, 7, and 9 GW/cm^2^.

This study introduces a refined finite element simulation approach to analyze the LSP process on 304L stainless steel with a focus on optimizing residual stress distribution and surface deformation. A key point in this work is the implementation of a triangular pressure pulse to more accurately mimic the real-time dynamic nature of laser-induced shockwaves. The simulation model is further strengthened by direct validation against experimentally measured residual stress profiles, enhancing its practical reliability. Additionally, the study provides a comprehensive parametric analysis by varying both laser power density and overlap ratio, two critical factors that jointly influence subsurface stress fields and surface morphology. This integrated modeling framework offers valuable insight into the control of residual stress through LSP and establishes a pathway for optimizing surface treatment parameters in structural applications of stainless steel.

While several studies have investigated LSP modeling in different materials, relatively few have focused on 304L stainless steel, particularly in welded conditions or critical structural applications like nuclear waste canisters [8,21,22,23]. Moreover, most existing models rely on rectangular or Gaussian pressure pulses that do not fully capture the actual dynamic nature of shockwave propagation. In this study, a triangular pressure pulse is used to more accurately simulate the physical profile of the laser-induced pressure. Additionally, the effect of overlap ratio (30% vs. 50%) and high-power laser densities (up to 9 GW/cm^2^) are analyzed in combination, providing insight into the non-linear interaction between intensity and residual stress. Experimental validation is included to support the simulation’s realism.

These aspects make this study distinct by providing a systematically validated simulation model for SS 304L, capturing both stress evolution trends and microstructural implications, which are particularly relevant for SCC resistance in safety-critical applications.

## 2. Modeling Methods

**Figure 2:**
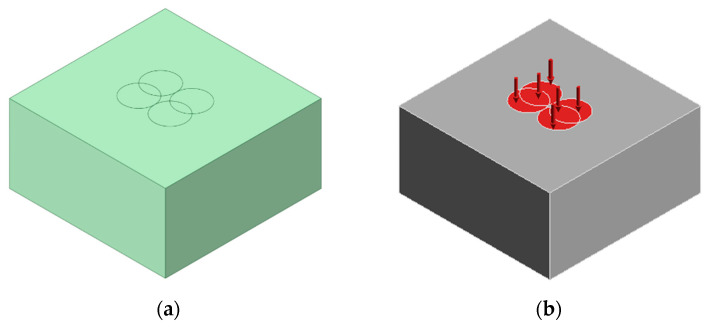
Input geometry for LSP simulation: (**a**) Arrangement of laser spots. (**b**) Application of pressure on the defined laser spots.

In this study, LSP simulations are conducted using ANSYS Workbench 2021. The 3D model has dimensions of 5 × 5 × 2.5 mm^3^. A general-purpose linear element order is employed to obtain slow and smooth transition of forces and resulting stress. A 3D solid element available in ANSYS Workbench 2021 was used in the analysis, specifically designed for continuum displacement and stress calculations. Table 1 gives the input geometry parameters used for simulation. Figure 2 illustrates the input geometry for LSP, highlighting both the laser spot arrangement and pressure application.

The meshing of the 3D model is coarse across the entire block, but a finer mesh is used at the laser shock spot. The mesh target value is 0.05 mm. At the top, however, the surface refinements feature is used to form a high density of elements. The Johnson–Cook (J-C) model is used, with thermal terms neglected, so the final loading is represented as pressure calculated using the relevant equations. A centrosymmetric pressure load with uniform distribution is applied to the 3D model and triangular-shaped pressure pulse is used for loading.

To ensure the accuracy and reliability of the simulation results in capturing the high strain rate and stress gradients induced by Laser Shock Peening (LSP), a non-uniform mesh was generated using unstructured 3D tetrahedral elements in ANSYS Explicit Dynamics. The mesh was refined in the top surface zone, where the laser pulse interacts with the material and where stress gradients are the steepest. In this laser-affected region, the element sizes ranged from 0.03 mm to 0.05 mm, while the bulk interior was meshed more coarsely with elements up to 0.1 mm in size. This graded mesh strategy allowed for high resolution near the impact zone while maintaining computational efficiency in less critical regions.

A mesh convergence study was performed by varying the average element size within the refined surface region, as this zone dominates the accuracy of stress and plastic strain predictions. Three refinement levels were tested: coarse (0.1 mm), medium (0.05 mm), and fine (0.03 mm). The peak von-mises stress was used as the convergence criterion. Results showed that the peak stress increased from 0.585 GPa (coarse) to 0.639 GPa (fine), with a difference of only 3.2% between the medium and fine mesh, indicating convergence. Based on this, the 0.05 mm element size in the laser-affected region was selected for all simulations. Detail comparison of mesh conversion study is given in Table 2.

**Figure 3:**
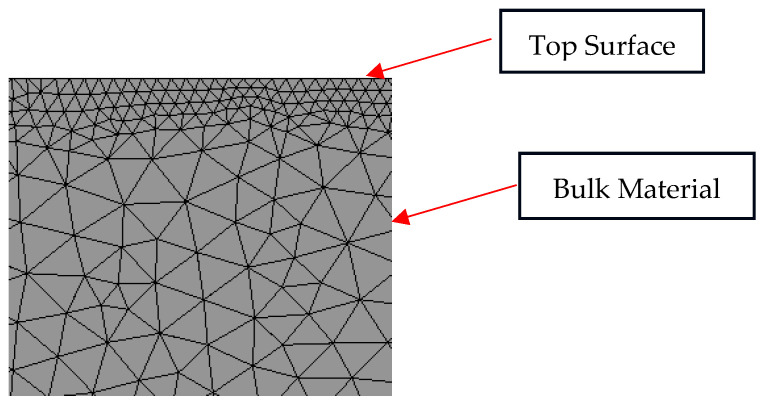
Mesh visualization for LSP processing.

Mesh quality metrics such as aspect ratio and skewness were verified, and no distorted or inverted elements were observed. Additionally, the refined surface region included at least 5 to 8 elements through the depth of the laser penetration zone to accurately capture shock propagation. The overall mesh structure with surface refinement and a gradual transition to coarser elements is shown in Figure 3, which highlights the critical resolution applied near the top surface. These mesh design considerations ensure sufficient fidelity for capturing residual stress distribution and validating simulation accuracy under high-energy LSP conditions.

In the simulation setup, the bottom face of the 3D block was fully constrained in all directions to mimic the fixed boundary condition during LSP. The other faces were left free to allow realistic wave propagation and deformation. For time stepping, ANSYS Explicit Dynamics automatically applied a variable time step based on the Courant–Friedrichs–Lewy (CFL) condition to ensure numerical stability. The total simulation time was set to 200 ns, which is sufficient to capture the complete shockwave propagation and residual stress stabilization within the material. The laser beam is modeled as a time-dependent pressure pulse with a triangular profile, rising to peak pressure in 5 ns, holding for 5 ns, and decaying over the next 10 ns for a total duration of 20 ns. This surface pressure mimics the peak stress caused by plasma expansion in a confined environment. A refined mesh of 0.05 mm element size was used in the laser interaction zone, with coarser meshing elsewhere. Boundary conditions included fully constrained nodes at the bottom surface and free boundaries elsewhere to allow natural stress wave propagation.

In this type of pulse, pressure rises rapidly in the first few nanoseconds and then decreases. Once the model is prepared with all material properties, geometry, and loading conditions, the experimental conditions acquired from the literature are considered to verify the consistency of the simulation results predicted by the developed model.

### 2.1. Material Model

Material model selection is a crucial step in simulation. The accuracy of output depends on how the material model accurately responds to high strain rates. An ideal material model should consider both thermal softening and strain hardening components of the material and their behavior under high strain-rate loadings. Several material models have been developed by researchers and validated against real-time yield data, including the Johnson–Cook (J-C) [21], Khan–Huang–Liang (KHL) [24], and Zerilli–Armstrong (ZA) models [25]. Among these, the J-C model is particularly effective for describing plastic deformation, and it has been adopted for use in the present study. The flow stress in the J-C model is expressed as follows:

(1) $$\sigma =\left[A+B\varepsilon_{p}^{n}\right]\left[1+C\;In{\dot{\varepsilon }}^{*}\right]\left[1-\left(T^{*}\right)m\right]$$

(2) $$T^{*}=(T_{Test}-T_{Room})/(T_{Melt}-T_{Room})$$

(3) $${\dot{\varepsilon }}^{*}=\dot{\varepsilon }/{\dot{\varepsilon }}_{0}$$

where σ and ε represent the flow stress and effective plastic strain, $\dot{\varepsilon }$ and ${\dot{\varepsilon }}_{0}$ denote the effective plastic strain and effective plastic strain rate. For temperature equation, T_Test_, T_Room_, and T_Melt_ stand for test temperature, room temperature, and melting temperatures, respectively. The constants A, B, C, m, and n are experimentally determined. Material properties for SS 304L are given in Table 3. The constant values listed in Table 4 correspond to the J-C material model, which describes the flow stress of materials under high-strain, strain-rate, and temperature conditions. In this model, the initial yield stress (A) defines the starting point of plastic deformation, while the strain hardening parameter (B) determines how the material strengthens as deformation progresses. The strain-rate sensitivity coefficient (C) accounts for the material’s response to different strain rates ($\dot{\varepsilon }$) while (ε_p_) represents the plastic strain, and *n* is strain hardening exponent in the equation. Thermal softening behavior (m) is controlled by an exponent that describes how the material weakens at elevated temperatures. Additionally, the melting temperature and reference temperature are used to normalize the thermal effects. These parameters are essential for accurately modeling material behavior in high-strain processes like LSP, where extreme strain rates and thermal effects significantly impact the mechanical response. Table 3 provides the J-C model constant values, which are taken from published experimental data [26].

**Figure 4:**
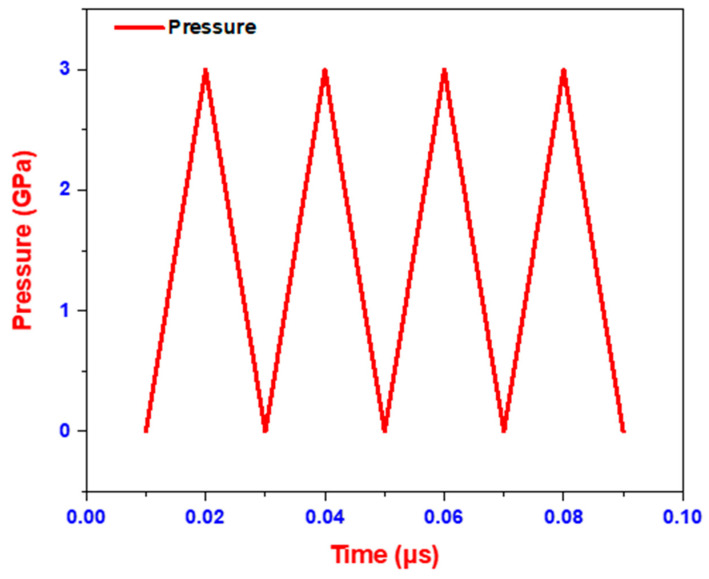
Laser pressure pulse vs. time plot.

The pressure wave generated by the laser shock has a duration that is three times longer than the laser pulse. The pressure profile used in this study is shown in Figure 4. The shock wave produced by the laser pulse consists of both pressure and heat. Since LSP is considered thermally isolated, the thermal terms in the J-C material model are neglected. This assumption is based on the fact that LSP exhibits a high strain rate and ultra-short duration process (typically in the order of nanoseconds), during which thermal diffusion is minimal due to the confined medium (water) that rapidly quenches the surface. Multiple studies have shown that under such adiabatic conditions, the mechanical response is dominated by pressure-induced plastic deformation rather than thermal softening [4,5]. Therefore, neglecting the thermal terms in the Johnson–Cook model is a valid simplification for capturing the primary mechanical effects of LSP in this context [22]. Assuming a uniform power density on the ablation layer with water as the confined medium, the peak pressure P is given as:

(4) $$P\left(GPa\right)=0.01\sqrt{\frac{\alpha }{2\alpha +3}}\sqrt{z}\sqrt{I_{0}}$$

where 1/z = 1/$z_{1}$ + 1/$z_{2}$. The terms $z_{1}$ and $z_{2}$ refer to the shock impedance of the confinement medium and the target material, respectively, measured in g·cm^−2^·s^−1^. $I_{0}$ denotes the laser power density, measured in GW·cm^−2^. α is the absorption coefficient, which is the material-dependent factor that defines how the laser energy is absorbed by the material or the confining medium. The peak pressure generated by the laser source should be 2–3 times greater than the Hugoniot Elastic Limit (HEL) of the target material, which helps prevent inverse plastic deformation.

(5) $$HEL=\frac{1-\upsilon }{1-2\upsilon }DYS$$

The material HEL can be calculated using Poisson’s ratio (υ) and dynamic yield strength (DYS) of that material.

### 2.2. Design of Simulations

A comprehensive study was conducted using a Design of Experiments (DOE) approach for simulation, focusing on the critical variables of spot overlap and laser intensity to evaluate their effects on residual stress and surface distortion during the LSP process. In the simulation work, two spots are considered in the overlap condition, with overlap values of 30% and 50%, combined with four laser intensities, 3, 5, 7, and 9 GW/cm^2^. These specific values were selected based on findings from previously published experimental studies [7,8]. These values demonstrated similar conditions and produced reliable results. The reason behind taking 30% and 50% overlap in consideration was to assess the impact of varying degrees of spot overlap on the uniformity and depth of compressive stress distribution. Given a fixed laser spot diameter of 1 mm, the 30% overlap corresponds to a center-to-center spacing of 0.7 mm, while the 50% overlap corresponds to 0.5 mm, ensuring a controlled comparison of overlap effects under consistent geometric conditions. Smaller overlap percentages often result in non-uniform stress fields, while larger overlaps can lead to more consistent and deeper compressive layers. In this current work, simulation parameters were modeled based on Nd:YAG lasers with a wavelength of 1064 nm and typical pulse durations of 8–10 ns. The input power densities ranged from 3 to 9 GW/cm^2^, corresponding to laser pulse energies in the range of 5–20 J depending on spot size and pulse duration.

In terms of laser intensity, the chosen values have a broad range. This allows us to investigate how different energy levels influence the extent of plastic deformation and residual stress within the material. Higher laser intensities are expected to generate more intense shock waves, potentially leading to greater plastic deformation but also increasing the risk of surface damage. The values were directly influenced by the parameters used in the experimental work, ensuring that our simulation setup is aligned with real-world conditions and allowing for a meaningful comparison between the simulated and experimental results. This approach not only strengthens the validation of our simulation but also enables us to refine our understanding of the relationship between laser parameters and material response, ultimately contributing to the optimization of LSP processes.

### 2.3. Boundary Conditions and Load Application

Boundary Conditions: The bottom face of the 3D block (5 × 5 × 2.5 mm^3^) was fully constrained in all translational and rotational degrees of freedom (i.e., Ux = Uy = Uz = 0; Rx = Ry = Rz = 0). This simulates the rigid support typically present in experimental setups during LSP. All other surfaces were free to deform, allowing realistic propagation of stress waves.

Pressure Load Application: A triangular pressure pulse was applied over a circular area of 1 mm diameter to simulate the effect of a laser pulse. The pressure was applied to the top surface along the Z-direction (normal to the surface). The pulse rises linearly to the peak pressure in 5 ns, holds for 5 ns, and falls linearly back to zero over the next 10 ns, giving a total pulse duration of 20 ns.

Peak Pressure Values: Based on laser power density values of 3, 5, 7, and 9 GW/cm^2^, the peak pressures were calculated using Equation (4), as described earlier. For stainless steel 304L with water as the confinement medium (shock impedance: 1.65 × 10^6^ g/cm^2^·s), the peak pressure for each laser power density was estimated using impedance matching relations (Equation (4)) and assuming an absorption coefficient (α) of 0.9. These calculated pressures were then used as input for the triangular pressure pulse applied in the simulation.

Time Step and Total Time: A variable time step was automatically determined by the solver using the Courant–Friedrichs–Lewy (CFL) condition. The total simulation time was set to 200 ns, sufficient for full stress wave propagation and residual stress stabilization.

Meshing Information (Reinforced): The mesh consisted of unstructured tetrahedral elements with a refined region on the top surface using element sizes of 0.03–0.05 mm and a coarser mesh (up to 0.1 mm) used in the bulk. The refined region had at least 5–8 elements through the depth of laser interaction.

Material Behavior: The Johnson–Cook material model was used with the constants specified in Table 3. Thermal terms were neglected due to the adiabatic nature of the high strain-rate process. No erosion, element deletion, or temperature coupling was activated.

### 2.4. Modeling Approximations and Their Impact

In this simulation framework, the interaction between the laser pulse and the ablative absorbing layer (e.g., black tape or aluminum foil) was not modeled explicitly. Instead, the laser–material interaction was simplified as an externally applied pressure pulse with a triangular time profile, which is a commonly accepted approach in finite element modeling of LSP [21,22,23]. This approximation assumes that the rapid plasma formation and expansion caused by ablation translates directly into a pressure wave on the workpiece surface.

The decision to omit the ablation and thermal conduction effects was based on the following considerations: (a) The thermal effects are minimal during LSP due to the ultra-short pulse duration (8–10 ns) and the presence of a confining water layer, which rapidly quenches surface heating. (b) The shockwave formation occurs within nanoseconds, and its mechanical effect dominates material deformation, especially for metallic systems like SS 304L. Previous experimental and simulation studies have validated this pressure-based simplification for high strain-rate modeling of LSP, with reasonably accurate stress predictions [12,13,14,15,16,17,18,19,20,21,22]. However, this approach neglects local thermal softening, material evaporation, and plasma kinetics, which may cause (a) slight overestimation of compressive stress magnitude and (b) loss of detailed insight into the heat-affected zones or melting thresholds. These limitations were partially mitigated by calibrating the pressure pulse magnitudes based on literature values (2–3× the Hugoniot elastic limit of SS 304L), ensuring physically realistic stress levels. The validation against experimental stress profiles further supports the appropriateness of this simplification for evaluating mechanical stress fields induced by LSP.

## 3. Results and Discussion

**Figure 5:**
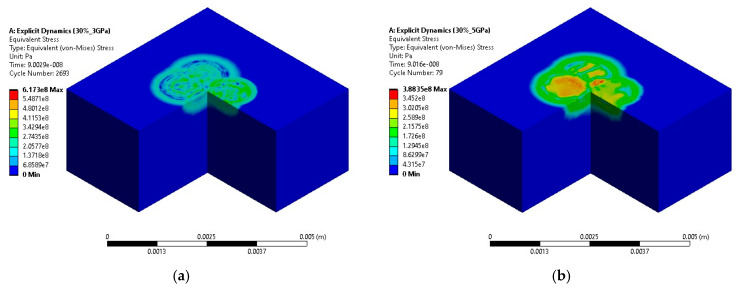

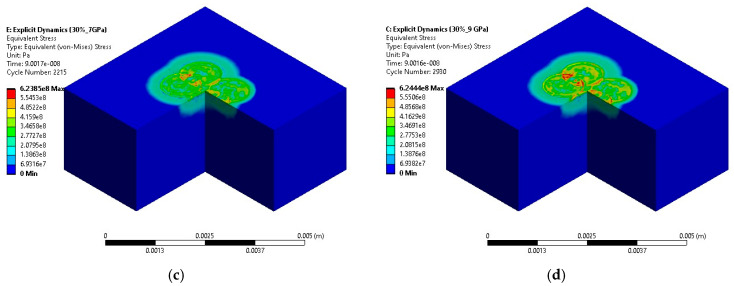
Residual stress contours for laser powers of (**a**) 3 GPa, (**b**) 5 GPa, (**c**) 7 GPa, and (**d**) 9 GPa with 30% overlapped LSP simulations.

As detailed in Section 2.2, simulations were performed under varying conditions to study the effects of LSP. We first considered a scenario with a 30% spot overlap and a spot diameter of 1 mm, varying the laser intensities at 3, 5, 7, and 9 GW/cm^2^. The observed maximum residual stresses were 0.62 GPa, 0.39 GPa, 0.62 GPa, and 0.63 GPa, respectively. These findings illustrate a non-linear relationship between laser intensity and residual stress, indicating that stress levels do not increase proportionally with increased intensity (Figure 5). In line with these observations, Jingyi Zhao et al. [27], in their experimental study on titanium alloys, reported similar non-linear behavior in residual stress responses with varying laser intensities.

The contour plots demonstrate the variation in stress distribution as laser power changes, providing insights into how laser energy impacts the material’s stress state. Comparative studies such as those by Zhaoru He et al. [28] also observed that increased spot overlaps lead to more homogeneous stress distributions, particularly in high-ductility metals such as aluminum. Their experimental results align closely with our simulations, reinforcing the applicability of increased overlaps for achieving desired mechanical properties across different materials

**Figure 6:**
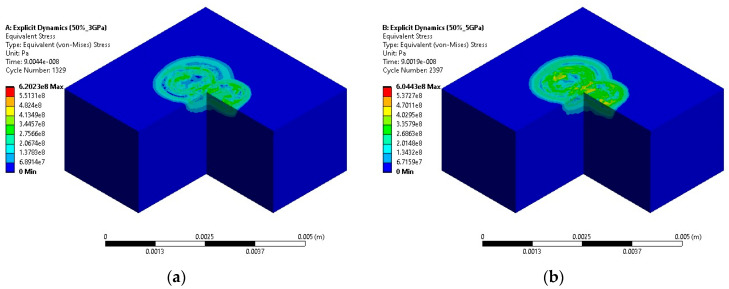

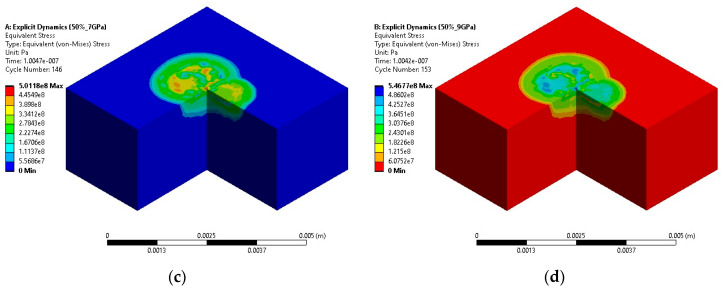
Residual stress contours for (**a**) 3 GPa, (**b**) 5 GPa, (**c**) 7 GPa, and (**d**) 9 GPa laser power with 50% overlapped LSP simulations.

Further simulations were conducted with a 50% spot overlap, maintaining the same spot diameter and varying the laser intensities similarly. As shown in Figure 6, maximum residual stresses measured were 0.62 GPa, 0.60 GPa, 0.50 GPa, and 0.55 GPa for the 3, 7, 5, and 9 GPa pressure intensities. This set of results highlights a more consistent stress profile at lower intensities, with only slight variations at higher intensities, underscoring how spot overlap significantly influences stress distribution, as shown in Figure 6.

### 3.1. Comprehensive Stress Profile Analysis

**Figure 7:**
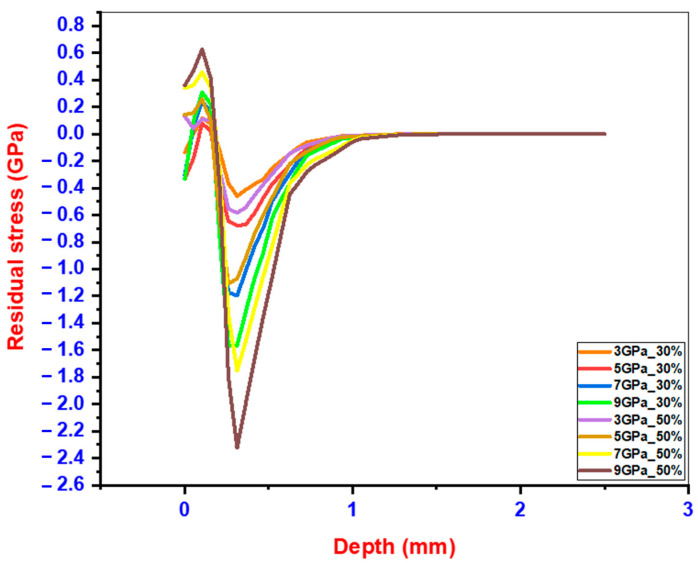
Residual stress distribution along depth following LSP treatment simulation.

The detailed stress profiles from all simulations are graphically represented in Figure 7, illustrating the impact of different overlap percentages. It was observed that a 50% overlap induces more residual compressive stresses compared to a 30% overlap. Additionally, it was noted that materials with higher yield stress are likely to retain higher magnitudes of compressive residual stress after LSP treatment. Further validating our results, Rohit Voothaluru et al. [29] in their longitudinal study on stainless steel subjected to LSP reported enhanced retention of compressive residual stresses with increased overlap percentages. Their experimental findings suggest that the effect of overlap is even more pronounced in materials with higher levels of inherent stress resistance.

The observed non-linear variation in residual stress with increasing laser intensity can be attributed to several physical mechanisms. At moderate intensities, higher laser energy produces stronger shockwaves that penetrate deeper into the material, inducing greater plastic deformation and enhancing compressive residual stress. However, beyond a certain threshold, further increases in intensity may lead to stress saturation or microstructural softening due to local thermal effects, even if not explicitly modeled. Additionally, the interaction of overlapping shockwaves can lead to complex wave superposition and reflection effects, which may either reinforce or partially cancel stress fields depending on their timing and location. These factors collectively contribute to the observed plateauing or reduction in residual stress at higher intensities, as also noted in previous experimental studies [27].

**Figure 8:**
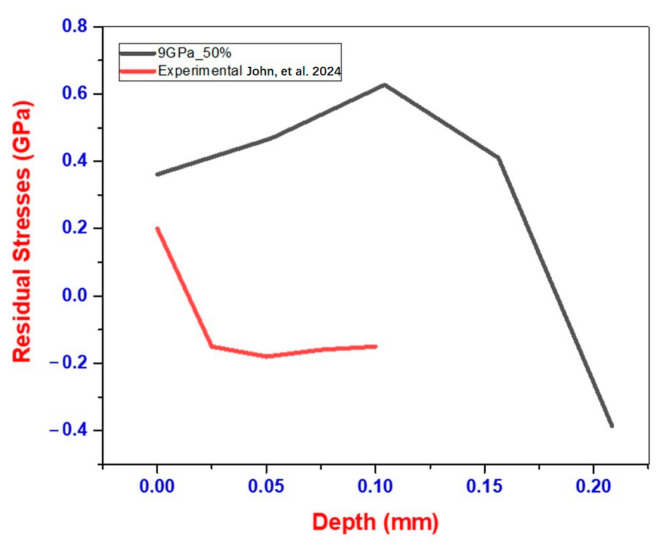
Comparison of simulated and experimental residual stress vs. depth profiles for a flat SS 304L surface after LSP (7 and 9 GW/cm^2^). While absolute magnitudes differ due to modeling simplifications and measurement limitations, the trend in surface tensile to subsurface compressive stress is consistently captured [8].

The transition from tensile to compressive stresses observed in the present simulations is corroborated by the experimental results reported in John et al. [8]. They examined the effects of LSP on stainless steel welds used in nuclear canister applications, without the application of any coatings. Their experimental setup utilized 7 and 9 GW/cm^2^ laser intensities, and they measured residual stresses using techniques such as X-ray diffraction and SEM. Notably, they reported a compressive stress curve pattern, which aligns with simulated outcomes shown in Figure 8. This validation emphasizes the accuracy and relevance of our simulation models for predicting the effectiveness of LSP in critical material applications. To validate our simulation results, we compared them with experimental findings by John et al. [8], as illustrated in Figure 8.

To validate the simulation outcomes, comparisons were made with experimental studies focusing on LSP effects on stainless steel. Flores-García et al. [30] conducted an experimental investigation on 304 stainless steel specimens that were laser-cladded with a cobalt-based alloy and subsequently subjected to LSP. Utilizing the contour method for residual stress measurement, they observed significant compressive residual stresses induced by LSP, which correlated with a 14% enhancement in fatigue life. These findings are consistent with our simulation results, particularly regarding the depth and magnitude of compressive stresses achieved through varying overlap ratios and power densities.

Additionally, a study by Li et al. [31] examined the microstructural changes in 17-7PH stainless steel welded joints post-LSP treatment. Their experimental results demonstrated that LSP promotes grain refinement and induces phase transformation from residual austenite to martensite, leading to improved mechanical properties. The observed increase in surface hardness and the development of compressive residual stress aligns with the trends predicted by our finite element model, thereby reinforcing the validity of our simulation approach. These experimental validations underscore the reliability of our simulation framework in accurately predicting the residual stress distributions and microstructural evolutions resulting from LSP in stainless steel materials.

### 3.2. Discussion on Discrepancy Between Simulation and Experimental Results

While Figure 8 illustrates the comparison between simulation and experimental residual stress profiles, a noticeable discrepancy is observed in the magnitude of compressive stress. The simulation predicts a peak residual compressive stress of approximately 0.62 GPa, whereas the experimentally measured value using X-ray diffraction (XRD) reaches only ~0.45 GPa. This ~27% difference can be attributed to several factors: (a) idealized assumptions in the FEM model, such as perfectly uniform pressure distribution, homogeneous material behavior, and no microstructural gradients; (b) neglecting thermal effects and ablative layer interactions, which may lead to overestimation of peak stress in pressure-only models; (c) limitations of XRD, including limited penetration depth, surface roughness influence, and peak broadening, which often underestimate near-surface stress levels.

Despite the discrepancy in absolute stress values, the qualitative trend matches well—both simulation and experiment show a transition from tensile stress at the surface to compressive stress at depth (~200–300 µm). This agreement in stress distribution pattern indicates that the simulation effectively captures the primary deformation and stress propagation mechanisms induced by LSP. Furthermore, the use of validated Johnson–Cook material parameters, mesh convergence checks, and triangular pressure profiles strengthen the physical reliability of the model.

Future work could incorporate thermomechanical coupling, grain orientation effects, and stochastic variability in laser parameters to improve numerical experimental agreement.

While the simulated residual stress profiles show a strong qualitative agreement with experimental trends reported in John et al. [8], certain discrepancies in stress magnitude and gradient are observed. These differences can be attributed to a variety of experimental and computational error sources that are inherent in LSP characterization and modeling.

From the experimental standpoint, X-ray diffraction (XRD), the most commonly used method for residual stress measurement, is subject to limitations such as penetration depth sensitivity, instrument calibration, and surface roughness artifacts, all of which may skew stress values, particularly near the top few microns. Additionally, local variations in grain orientation (texture) and microstructural inhomogeneities especially in LSP-treated zones can lead to stress measurement scatter due to anisotropic strain responses. Measurement uncertainty is also introduced by instrumental broadening, peak shifting, and subsurface plasticity, particularly in multi-pass or overlapping laser peening applications.

On the simulation side, idealized assumptions made in the FEM models such as perfectly uniform pressure loading, homogeneous material behavior, and absence of microstructural effects, may also contribute to quantitative deviations. The use of a Johnson–Cook model without thermal coupling simplifies the actual thermomechanical response of the material, particularly under high laser intensities. Furthermore, boundary condition constraints and mesh resolution near the surface critically affect the accuracy of stress gradients, especially in the first 200–300 µm.

It is also important to note that residual stress is a path-dependent outcome, influenced by the entire deformation and recovery history of the material, which may not be fully captured in single-step or uncoupled simulations. Despite these limitations, the observed trends in stress evolution, such as the transition from near-surface tensile to subsurface compressive stress match experimental profiles well, validating the physical fidelity of the model for process optimization studies.

In addition to inducing residual stress, LSP is known to cause significant microstructural alterations, particularly in austenitic stainless steels such as SS 304L. High strain rates and associated plastic deformation from LSP lead to localized grain refinement, formation of deformation twins, and increased dislocation density near the surface [1]. These changes contribute to mechanical strengthening, improved fatigue resistance, and enhanced wear and corrosion behavior. Although this study focuses on continuum-scale stress response using FEM, it is recognized that these microstructural modifications influence the material’s constitutive behavior. Previous studies have demonstrated that such effects are prominent within the first few hundred microns below the surface, aligning with the depth range of compressive stress observed in our simulation [3,4]. Incorporating microstructure evolution models such as dislocation density-based hardening or crystal plasticity frameworks could provide deeper insight into the coupling between residual stress and microstructural features.

### 3.3. Microstructural Evolution

LSP is a high strain-rate surface modification technique that induces Severe Plastic Deformation (SPD) and results in complex microstructural changes, particularly in austenitic stainless steels such as SS 304L. These microstructural alterations such as dislocation multiplication, grain refinement, and phase transformation are critical for enhancing surface mechanical properties and resistance to Stress Corrosion Cracking (SCC). While the present work is focused on modeling of LSP, we validate our simulation findings by comparing them to the experimental results from our work reported in [8], where comprehensive microstructural and mechanical characterization of SS 304L subjected to LSP without protective coating (LSPwC) was investigated.

In our study, LSPwC was performed at varying power densities (7 and 9 GW/cm^2^) and overlap ratios (30% and 50%), producing a substantial depth of plastic deformation and compressive residual stresses. X-Ray Diffraction (XRD) data revealed the formation of α′-martensite (strain-induced) at higher intensities, while Full Width at Half Maximum (FWHM) analysis showed significant broadening, indicating increased dislocation density and refined subgrain structure.

Our finite element model, which incorporates a triangular pressure pulse and material parameters calibrated for SS 304L, predicts a peak compressive residual stress of approximately 0.62 GPa located at ~250 µm beneath the surface. This value is in close agreement with our experimental results shown in Figure 8. The simulated stress gradient also mimics the experimental trend, where surface residual tensile stresses are replaced by deep compressive zones due to shock wave propagation and plastic strain localization.

Furthermore, the experimental FWHM profiles reported from our work showed maximum broadening within the first 100 µm of the surface, gradually stabilizing in the 150–300 µm range. This observation correlates with our stress distribution, indicating that these regions are subject to high levels of plastic deformation. The grain refinement effect, inferred from FWHM and confirmed via SEM in their study, is consistent with the simulated plastic strain concentration near the top layers in our model.

Another important aspect observed experimentally is the phase transformation from face-centered cubic (FCC) austenite to body-centered tetragonal (BCT) α′-martensite under LSP-induced strain. This transformation is thermodynamically driven by high local strain and strain rates exceeding 10^6^ s^−1^, as typically achieved in LSP. While our simulation does not explicitly model phase transformation, the magnitude and gradient of von-mises stress in the surface layers (exceeding 800 MPa locally before relaxation) suggest that transformation thresholds are likely met, particularly under higher simulated power densities.

**Figure 9:**
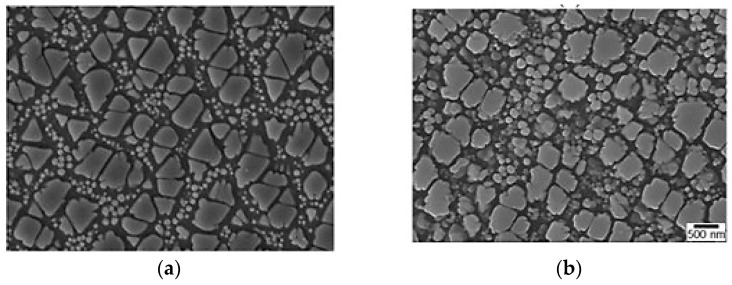
SEM images showing microstructural evolution of SS 304L: (**a**) without LSP treatment, (**b**) with LSP treatment. Scale bar: 500 nm [32]. Both images were recorded under the same magnification.

To visually support the microstructural changes resulting from LSP-induced plastic deformation, Figure 9 presents SEM images showing (a) untreated SS and (b) laser-treated SS samples, which shows the evolution of grain morphology under laser peening intensity or exposure. As reported by Liu et. al. [32], the untreated surface exhibits angular and less uniform grains, while the laser-peened surfaces show significant grain refinement, uniformity, and nanoparticle formation. These microstructural refinements reflect higher local dislocation density and dynamic recovery mechanisms activated during severe plastic deformation. The transition to more equiaxed and refined grains correlates with regions of high von-mises stress observed in our simulation. This image-based evidence reinforces the role of LSP in enhancing surface integrity and mitigating crack initiation sites, which supports the observed improvements in SCC resistance and residual stress behavior.

From a mechanistic viewpoint, the synergy between residual stress, plastic strain, and microstructural evolution is critical for improving SCC resistance. Our prior work [8] demonstrated that LSPwC-treated U-bend specimens exhibited delayed crack initiation and suppression of crack propagation even after exposure to boiling MgCl_2_, with SEM revealing crack arrest near the subsurface layer (~20 µm) where compressive stress and refined grains dominate. Our stress simulations show that the compressive stress regime extends well into this depth range, supporting the hypothesis that our model successfully captures the mechanically affected zone responsible for crack retardation. The technical consistency between our numerical predictions and the experimental findings by our prior work [8] enhances the credibility of our simulation model. Despite the absence of direct microstructural imaging in this study, the use of validated data from the peer-reviewed literature bridges the gap and reinforces the interpretation of the finite element results. Future integration of microstructure-sensitive constitutive models such as crystal plasticity or dislocation-based hardening models would further improve the accuracy of predicting phase transformation and grain evolution in LSP-treated components.

## 4. Conclusions and Future Directions

This study contributes new insights into the effect of laser power and overlap ratio on residual stress development in SS 304L stainless steel using a finite element approach. The use of a triangular pressure pulse, which better approximates real-time LSP pressure dynamics, along with a validated material model (Johnson–Cook) and fine mesh convergence analysis, enhances the model’s physical fidelity. By simulating both 30% and 50% overlaps across a range of laser intensities (3–9 GW/cm^2^) and comparing the results with experimental data, the model provides a comprehensive analysis of LSP parameter interactions, including non-linear stress trends and practical stress limits. This approach is particularly novel for weld-joint applications in nuclear environments where predictive accuracy of surface stress fields is critical. The results revealed that the relationship between laser intensity and residual stress is not strictly linear. Notably, the highest compressive stresses were observed at specific intensities—particularly at 7 and 9 GW/cm^2^—indicating the presence of an optimal intensity range where the material response is most effective. Increasing the spot overlap from 30% to 50% generally led to deeper and more uniform compressive stress profiles, which is advantageous for applications requiring improved fatigue resistance or enhanced mechanical performance over larger surface areas. A comparison between simulation and experimental data showed good agreement in terms of overall trends, though some variation in stress magnitude was observed. These differences are expected, given the idealized nature of simulation models and the complexity of real-world measurements. Overall, the findings contribute to a deeper understanding of LSP process parameters and offer practical insights for optimizing treatment strategies in industrial applications.

These insights are particularly valuable for high-performance applications such as aerospace turbine blades, nuclear canister welds, and marine structural components, where enhanced fatigue life and corrosion resistance are critical. For future work, thermomechanically coupled simulations could be conducted to better capture the role of temperature in stress evolution. Additionally, experimental validation using advanced characterization techniques such as neutron diffraction, Digital Image Correlation (DIC), or microhardness profiling could further strengthen the simulation results and enable real-world implementation.

## Figures and Tables

**Table 1:** Geometry input features for LSP simulation.

Parameter	Unit	Value
Material	-	Stainless Steel 304L, Annealed
Block dimension	mm	5 × 5 × 2.5
Laser spot diameter	mm	1
Number of laser spots	Count	4

**Table 2:** Mesh convergence results based on von-mises stress at 30% overlap, 3 GPa input.

Mesh Type	Element Size (mm)	Maximum Von-Mises Stress (GPa)	Stress Difference from Finer Mesh (%)
Coarse Mesh	0.1	0.58	8.20%
Medium Mesh (used)	0.05	0.61	3.30%
Fine Mesh	0.03	0.63	—

**Table 3:** J-C model constant values [26].

A	B	n	C	m	$\dot{\varepsilon }$	T_m_	T_r_
452	694	0.311	0.0067	0.996	0.001	1673 K	273 K

**Table 4:** Stainless steel 304L material properties.

Young’s Modulus (GPa)	Poisson Ratio	Density (kg/m^3^)
195.1	0.27	7904

## Data Availability

The original contributions presented in this study are included in the article. Further inquiries can be directed to the corresponding author.
